# Surgery for post-traumatic hydrocephalus: lessons, challenges and future directions

**DOI:** 10.3389/fneur.2026.1808233

**Published:** 2026-04-10

**Authors:** Niccolò Neri, Arianna Vignaroli, Giorgio Palandri

**Affiliations:** 1 Department of Neurosurgery, Istituto delle Scienze Neurologiche di Bologna IRCCS Ospedale Bellaria, University of Bologna, Bologna, Italy; 2 Department of Neurosurgery, Istituto delle Scienze Neurologiche di Bologna IRCCS Ospedale Bellaria, Bologna, Italy

**Keywords:** cranioplasty, decompressive craniectomy, neurotrauma, post-traumatic hydrocephalus, shunt, valve

## Abstract

**Introduction:**

Post-traumatic hydrocephalus (PTH) is characterized by ventriculomegaly, intracranial pressure (ICP) impairment and progressive neurological deterioration; it is a common yet often under-recognized and under-treated complication of traumatic brain injury (TBI). Early identification and intervention are critical for optimizing neurological recovery and functional outcomes. The proportion of patients requiring intervention for PTH is highly variable but is supposed to reach up to one-third of individuals sustaining a TBI. Shunt surgery represents gold standard treatment, but precise recommendations regarding therapeutic decision-making and operative techniques are still lacking. The aim of this narrative review is to synthetize current evidence on surgical management of PTH, highlighting available options with their respective strengths and limitations.

**Methods:**

A comprehensive literature search was conducted focusing on studies from the past decades that reported surgical management of PTH. Relevant retrospective and prospective series, comparative analyses, and recent narrative/systematic reviews were included.

**Discussion:**

Ventriculoperitoneal shunting (VPS), lumboperitoneal shunting (LPS), and ventriculoatrial shunts (VAS) are the most widely explored techniques in PTH management. VPS is the most performed treatment, but LPS and VAS are feasible alternatives showing similar rate of improvement although possibly higher risks of malfunction and systemic complications should be considered. Programmable valves represent the preferred choice for PTH shunt surgery, demonstrating less complications and need of surgical revisions compared to fixed-pressure systems. ETV—traditionally viewed as a relative contraindication in PTH—has shown satisfactory results, though long-term efficacy remains uncertain. Simultaneous cranioplasty and shunting is increasingly reported in clinical practice, however there is contradictory evidence supporting its safety and efficiency. Moreover, outcomes and complications rate vary widely, reflecting the heterogeneity of patient populations, injury patterns, and timing of intervention. There is also limited but growing evidence for conservative strategies, particularly in long-term management of PTH and TBI’s clinical sequelae, even though their role is less clearly delineated.

**Conclusion:**

PTH management has deeply evolved during the last decades, enhancing the standard of care and achieving better long-term prognosis, but still lacks firm consensus on diagnostic and therapeutic indications, with scarce prospective comparative data. Refining surgical decision-making and prospective, multicenter trials are crucial to improve outcomes of this complex condition.

## Introduction

Post-traumatic hydrocephalus (PTH) is a debilitating sequela of traumatic brain injury (TBI) (1, 2). Characterized by disrupted cerebrospinal fluid (CSF) dynamics and uncertain pathophysiology, PTH severely compromises brain homeostasis (1, 3, 4), leading to cognitive decline, motor disturbances, and loss of independence (5, 6). Consequently, early identification and intervention are paramount to prevent delayed recovery and optimize long-term neurological outcomes (7, 8).

The reported incidence of PTH in TBI populations varies widely, with estimates ranging from less than 1% to one-third of the patients (7, 9, 10). This heterogeneity in the literature is attributable to several factors, including diverse patient population characteristics and varying methodological criteria across studies. A significantly higher proportion of patients develop PTH following decompressive craniectomy (DC) (5), a specific subgroup where incidence rates reach up to 50% (2, 7). Additionally, young age, severe clinical presentation with lower GCS at admission, and coexisting dural venous sinus injuries appear to be associated with an increased risk of requiring shunt surgery (7). Overall, shunt surgery for PTH is associated with substantial clinical improvement, particularly when intervention occurs within the first few months after TBI. However, outcomes and complication rates vary, as these patients are often fragile, presenting with high comorbidity rates and a wide heterogeneity of injury patterns (4).

In current practice, CSF diversion via ventriculoperitoneal shunt (VPS) remains the most prevalent treatment for PTH. Nevertheless, numerous other surgical options have been explored and reported as feasible alternatives, including ventriculoatrial (VA) and lumboperitoneal (LP) shunting, endoscopic third ventriculostomy (ETV), and simultaneous cranioplasty with CSF diversion (4, 9–13). Recent reports have also explored innovative techniques such as long-tunneled external ventricular drainage (LTEVD) (11). Furthermore, there is growing evidence supporting conservative strategies, particularly in the long-term management of PTH and TBI clinical sequelae (9, 14). Despite this, precise recommendations regarding therapeutic decision-making and operative techniques remain undefined. The aim of this narrative review is to synthesize current evidence on the surgical management of PTH, highlighting available options along with their respective strengths and limitations.

## Materials and methods

A structured search strategy was implemented across the PubMed and Scopus electronic databases for studies published between 2000 and 2026, utilizing a combination of Medical Subject Headings (MeSH) terms and Boolean operators (AND, OR). The primary search string incorporated keywords including, but not limited to: *post-traumatic hydrocephalus*, *PTH*, *shunt*, *ventriculoperitoneal shunt*, *ventriculoatrial shunt*, *lumboperitoneal shunt*, *cranioplasty*, *endoscopic third ventriculostomy*, and *ETV*.

Eligibility criteria were predefined to include original clinical series (prospective and retrospective), comparative studies, and high-impact reviews. The scope specifically addressed the surgical management of PTH—focusing on VPS, VAS, LPS, and ETV—as well as the role of concomitant cranioplasty, long-term functional outcomes, and conservative management strategies. While editorial comments, letters, and conference abstracts were excluded, case reports were selectively included if they addressed unconventional treatments with limited existing evidence. Landmark papers on iNPH and other hydrocephalus subtypes were also included to establish physiological and clinical benchmarks. The search was limited to articles published in English. The selection process involved an initial screening of titles and abstracts for relevance, followed by a rigorous full-text evaluation. Additionally, manual cross-referencing of retrieved bibliographies was performed to identify further pertinent studies. Data were qualitatively synthesized to delineate evolving surgical trends, prognostic factors, and current controversies in PTH management.

## Discussion

### Ventriculo-peritoneal shunt

Developed in the 1970s (15), VPS was initially regarded as inferior to ventriculoatrial shunting (VAS) due to higher rates of peritonitis and distal malfunction. However, it progressively gained favor following the recognition of severe and sometimes fatal VAS-related complications, such as cardiopulmonary embolism, cardiac insufficiency, bacteremia, nephritis, and renal failure (16–18). Over time, VPS became the standard surgical treatment for PTH (4, 6, 9, 19, 20).

Timely VPS placement has been demonstrated to substantially improve symptoms and neuroradiological features in PTH patients (4, 19, 21, 22). Kowalski et al. showed that earlier VPS placement is associated with a shorter duration of Post-Traumatic Amnesia (PTA), reduced rehabilitation length of stay, and overall better clinical outcomes (19). Similarly, Wang et al. reported that VPS led to clinical improvement in 71% of patients treated early, identifying older age, delayed treatment, and poor initial Glasgow Outcome Score (GOS) as predictors of inferior outcomes (22). However, early intervention is often hindered by the complex clinical presentation of TBI survivors. These patients often present with multisystemic sequelae that can mask specific signs of hydrocephalus, leading to diagnostic ambiguity. Furthermore, when PTH is suspected, the imperative to manage concomitant life-threatening injuries—such as major vascular dissection, acute respiratory distress or unstable fractures—often necessitates a therapeutic delay.

This systemic fragility also increases the technical complexity of shunt insertion and elevates the risk of postoperative adverse events, which are not uncommon (9, 23). The overall incidence of shunt revision following VPS is frequently reported to be high (12, 23). PTH patients carry a higher risk of shunt complications compared to those with other forms of hydrocephalus (e.g., iNPH); factors such as prolonged intensive care hospitalization and prior DC further exacerbate susceptibility to infection, hemorrhage, malfunction, and over-drainage (4, 24). To mitigate these events, recent evidence suggests that utilizing programmable valves or alternative shunting techniques (e.g., VAS, LPS) may reduce complication rates—particularly over-drainage and mechanical failure—thereby optimizing outcomes in this high-risk population (22, 25, 26).

### Ventriculo-atrial shunt

Following Gartner’s suggestion in 1896 to treat hydrocephalus by shunting CSF into the venous or lymphatic system of the head and neck, ventriculo-auriculostomy – in which the distal catheter drains CSF into the right atrium – became the first surgical technique implemented in clinical practice around the 1960s (27). VAS has long been considered a second-line treatment due to its association with severe complications, reserved for patients in whom VPS was contraindicated. However, recent technical advancements in surgical planning, fluoroscopy, and shunt design have proven effective in minimizing VAS-related risks (28), gradually leading to a re-evaluation of this procedure as a suitable and safe option.

The primary indication for prioritizing VAS over VPS in this population remains the high prevalence of concomitant abdominal injuries and scarring resulting from multisystemic trauma. These abdominal lesions may preclude the safe placement or long-term functioning of a peritoneal catheter, making the atrium an excellent alternative for CSF drainage (22). Furthermore, recent studies focusing on patients with various forms of NPH have demonstrated that VAS-related complications are highly uncommon in older patients and are not significant drivers of morbidity. McGovern et al. and Brenner et al. found the risk of serious systemic life-threatening complications (e.g., glomerulonephritis, heart failure, cardiopulmonary embolism) to be very low in VAS cohorts (29, 30). Additionally, lower rates of distal catheter obstruction and the need for surgical revision have been documented for VAS compared to VPS. Hung et al., in a retrospective cohort of 496 patients (150 VAS, 346 VPS), reported shunt obstruction rates of 5.3% vs. 17.6% and revision rates of 13.3% vs. 32.4%, respectively (31). Similarly, systematic reviews by Giordan et al. and Lo Bue et al. confirmed that VAS is associated with a lower risk of shunt dysfunction and reduced need for reoperation, with an infection and mortality profile comparable to VPS (32, 33). In McGovern et al.’s retrospective analysis, the distal catheter revision rate was 2.7% for VAS versus 6.6% for VPS, with nearly identical proximal catheter revision rates (2.7% vs. 2.5%), corroborating existing data. Another advantage is that VAS can be performed under local anesthesia, reducing operating times and complications related to drug administration (34), which may represent a significant benefit in fragile patients.

In summary, while VPS remains the standard treatment for PTH, VAS should be regarded as an effective alternative for post-traumatic patients rather than merely a salvage procedure. It is primarily indicated when abdominal injuries complicate peritoneal access, offering a safety profile comparable to VPS with the added benefit of potentially lower rates of mechanical failure and revision (4, 8, 24, 31).

### Lumbo-peritoneal shunt

LPS emerged in the mid-20th century as a minimally invasive CSF diversion technique restricted to communicating hydrocephalus, with early applications dating back over 60 years, anticipating widespread VPS adoption in the 1970s-1980s (35). LPS rapidly gained interest for iNPH treatment, challenging VPS dominance in Western practice due to surgeons’ familiarity (36). Unlike VPS and VAS, which require intracranial catheter placement and carry risks of parenchymal injury, LPS accesses the subarachnoid space via lumbar puncture. By avoiding brain penetration, this approach may offer substantial advantages in terms of reduced invasiveness and better patient compliance (23). Recent evidence from Seemann et al. emphasizes this benefit, noting that LPS represents a primary rescue strategy in patients with hydrocephalus and concomitant complex cranial wounds following neurosurgical procedures or direct trauma. LPS can favor wound healing and reduce the risk of recurrent infections while ensuring effective CSF diversion (37).

Although the choice between these procedures remains debated, many studies have documented similar results regarding outcomes, complications, and cost-effectiveness compared to VPS or VAS. Data from the SINPHONI trial (dealing with iNPH patients) and large meta-analyses indicate that LPS leads to clinical and neuroradiological improvements, statistically comparable to VPS (36, 38), with an even lower incidence of overall post-operative complications (12.98% vs. 23.8%), particularly regarding hemorrhages (2.4% vs. 5.03%), catheter obstruction/malfunction (3.99% vs. 8.31%), and infections (1.53% vs. 5.41%) (35). These benefits are remarkably significant in post-traumatic patients who are at high risk of multiple cranial complications, such as fractures, hemorrhages, or the need for DC, which may complicate an intracranial approach. Wang et al. studied 158 patients treated with LPS or VPS for post-hemorrhagic hydrocephalus (PHH), reporting that LPS matches VPS efficacy with 56% clinical (GCS increase ≥2) and 29% radiological (EI < 0.3) improvements versus 42 and 36%, respectively. They also confirmed that LPS reduces the risk of hemorrhage (1.78% vs. 13.72%) and the total complication rate (8.93% vs. 26.47%) (23). Furthermore, historical concerns regarding a higher percentage of over-drainage have been revised by recent studies demonstrating that the integration of a gravitational unit into LPS systems successfully mitigates postural pressure changes, resulting in a safety profile comparable to VPS (39).

LPS should therefore be recognized as a distinct, less invasive procedure serving as a standard of choice for patients with concomitant cranial injuries, and as a generally viable alternative with similar efficacy and complication rates when VPS or VAS are contraindicated. While VPS remains the standard approach across all hydrocephalus types, evidence strengthens the argument that shunt selection should be individualized based on patient characteristics and hydrocephalus etiology, as no clear differences exist in overall outcome and complication rates (9).

### “Unconventional” shunts

In rare circumstances where standard shunt placement proves unsuitable—scenarios particularly relevant to post-traumatic patients with extensive abdominal scarring, thoracic trauma, vascular injuries, cardiopulmonary complications or chronic infections—alternative drainage sites serve as critical salvage procedures. Ventriculopleural (VPL) shunt represents the most common alternative though its use is often limited by a high rate of failure related to pleural effusion and subsequent respiratory distress, particularly in patients with impaired resorptive capacity (40). Craven et al. in 2016 demonstrated that VPL shunts had a median survival of 14 months after which 54.5% were revised or removed, with an overall complication rates reaching 45%, primarily driven by pleural effusion, construct obstruction, and over-drainage (41). Evidence from mixed adult and pediatric cohorts suggests that while VPL shunts are effective, they are associated with a significant incidence of respiratory distress; a meta-analysis of 543 patients reported a 16% rate of pleural effusion, which remains the primary mode of failure (30). Despite these risks, VPL shunts remain a well-tolerated second option when VPS or VAS are contraindicated or have failed (42).

The ventriculogallbladder (VGB) shunt is an additional unconventional shunt, primarily documented in pediatric series and isolated adult case reports. Literature reviews indicate that these shunts remain patent and functional in approximately 60–70% of the patients, with infection and distal obstruction identified as the leading failure modes (43). Clinical series focusing on pediatric patients (mean age 6.1 years) highlight that VGB shunts can function effectively even after multiple ventriculoperitoneal shunt failures, though they require specific anatomical integrity of the biliary system (44). In adult salvage scenarios, VGB shunts have been successfully employed (45). The risk of mechanical failure includes rare but severe complications such as shunt fracture leading to bile peritonitis (46).

In extreme “last resort” cases, ventriculovesical (VVeS) shunts have been utilized. While providing immediate CSF diversion, they are burdened by significant urological morbidity, specifically the formation of bladder stones due to chronic foreign body irritation and the inherent risk of ascending urinary tract infections (47, 48). In conclusion, while these unconventional approaches provide life-saving alternatives when standard peritoneal or atrial sites are contraindicated, they are characterized by high complication profiles and substantial failure rates. Their use necessitates rigorous patient selection, specialized surgical expertise, and intensive long-term surveillance, reflecting the clinical complexity of managing hydrocephalus in patients with multiple competing anatomical constraints.

### Adjustable valves and anti-siphoning devices

The choice between an adjustable valve (AV) and a fixed-pressure valve represents a critical consideration in PTH management (26). AVs allow for non-invasive adjustment of opening pressure, enabling neurosurgeons to respond promptly to complications such as over-drainage or under-drainage, thereby reducing the need for surgical revision (9, 26)—a capability that proves particularly advantageous in PTH patients, where the frequent necessity of DC and the high risk of overdrainage-related complications present substantial clinical challenges (26). In a long-term follow-up analysis of adult patients with PTH provided by Chen et al., it was demonstrated that those treated with AVs had a significantly lower initial revision rate and superior revision-free survival compared to those with fixed valves. While cost-effectiveness and device availability remain issues in resource-limited settings, clinical evidence demonstrates that AVs substantially facilitate complication management and improve overall clinical outcomes in PTH patients, supporting their preferential selection where feasible (24, 26). These results were confirmed in NPH cohorts treated with AVs by Zemack et al., where valve adjustments in the immediate postoperative period were documented to improve clinical outcomes in 49.1% of cases, with larger adjustments (≥30 mm H₂O) proving more effective than minor ones. The primary mechanism underlying AV superiority in PTH appears to be the ability to achieve precise valve tuning within the first 6 months after implantation – a critical window for preventing over-drainage, which represents the most frequent complication in post-traumatic patients (25, 26).

Furthermore, this capability is particularly important in PTH patients who have undergone DC. In these cases, the absence of a bone flap complicates CSF dynamics, making adjustability necessary to avoid extra- and subdural collections. Additionally, we advocate for the implantation of adjustable valves in patients with DC, as they are crucial for optimizing the subsequent cranioplasty procedure. This technology offers the unique advantage of regulating ventricular dimensions perioperatively; specifically, by up regulating the setting before cranioplasty to reduce subdural and extradural collections and subsequently titrating the pressure to adapt to the patient’s post-cranioplasty hydrodynamic status. Comparative data from Vedantam et al. and Fotakopoulos et al. indicate that fixed-pressure valves are associated with a higher incidence of shunt dysfunction in patients treated with DC, reinforcing the theoretical advantage that non-invasive, prompt adjustment via AVs allows for superior CSF dynamic management (49, 50).

The selection of AVs involves diverse technologies that may impact the management of patients requiring a shunt. Distinct mechanical architectures are designed to facilitate non-invasive pressure modulation, such as the Integra Codman-Hakim^®^ stepping motor system, which utilizes a ruby ball on a flat spring adjusted via a spiral polyethersulfone staircase to provide 18 precise pressure increments. In contrast, other valves employ a magnetic rotor-cam mechanism to alter spring tension across five performance levels or integrates a sophisticated magnetic lock consisting of dual micro-magnets oriented in opposite directions to ensure setting stability against external interference. Furthermore, valves such as the Sophysa Polaris^®^ and Miethke proGAV 2.0^®^ incorporate a rocker or magnetic brake mechanism that is designed to resist spontaneous adjustments in MRI fields up to 3 Tesla, as the adjustment rotor is mechanically decoupled from the valve spring unless a specific programmer is applied. Similarly, the Codman CERTAS^®^ Plus utilizes a ball-and-socket magnetic brake to ensure setting stability. These aspects are crucial in shunted PTH patients who usually undergo frequent MRI during follow-up and long-term rehabilitation (51, 52).

The use of ASDs and gravitational units to mitigate posture-related complications also appears to be fundamental. ASDs are designed to counteract the “siphoning effect”—an excessive drainage of CSF caused by the hydrostatic pressure gradient when the patient assumes an upright position. Current literature documents that ASDs significantly reduce the incidence of over-drainage-related complications, such as subdural hematomas and hygromas, compared to differential pressure valves alone, without increasing the risk of a system malfunction (53, 54). Jakiela et al. highlighted that the integration of gravitational units effectively maintains physiological ICP across varying postures (55). This concept is supported by Miyake et al. and Sun et al., who documented that utilizing these devices, in both cranial and lumbar shunting, provides protection against both over-drainage and acquired Chiari malformations, ensuring that functional recovery is not impeded by postural ICP variations (56, 57).

The technical distinction between these technologies is further highlighted by their specific anti-siphoning mechanisms. While conventional membrane-based anti-siphon devices (ASDs)—such as the Delta^®^ valve—rely on subcutaneous pressure to counteract siphoning, they are prone to failures caused by external compression or tissue encapsulation (53). In contrast, gravitational devices utilize heavy tantalum or stainless-steelballs to increase the opening pressure only in the upright position, providing a more reliable and posture-dependent regulation of CSF flow (53). A comparative analysis within a simulated iNPH environment showed that flow-regulated devices demonstrate high precision in maintaining target drainage rates while upright, though they remain susceptible to overdrainage under conditions of slightly elevated intraperitoneal pressure. Conversely, adjustable gravitational units offer the advantage of patient-specific calibration for vertical posture but their efficacy remain contingent upon precise alignment with individual anatomical height and baseline abdominal pressure, as fluctuations in the latter can lead to significant drainage deviations. Finally, membrane-controlled systems provide the most robust prevention of siphoning by effectively halting flow in the upright position, but they fail to facilitate the continuous drainage necessary for physiological CSF diversion (58). Regarding clinical efficacy and safety, the multicenter SVASONA trial demonstrated that the use of proGAV^®^ with an integrated proSA^®^ gravitational unit significantly reduces the risk of overdrainage-related complications compared to non-gravitational programmable valves. Specifically, the study found that the rate of overdrainage-related complications was five times lower in the gravitational group compared to the programmable group without compromising the improvement in clinical symptoms (59).

The complexity of these fragile patients demands a versatile drainage system, favoring the use of AVs and ASDs as first-line treatment. PTH patients often present with severe neurological deficits initially implying prolonged bed rest, yet frequently undergo a rehabilitation pathway involving progressive postural changes aimed at the reacquisition of the upright position. Such drastic postural shifts create hydrodynamic challenges that fixed-pressure valves can hardly accommodate. AVs and ASDs help minimize complications requiring surgical revision and mitigate the deleterious effects of positional changes and uncontrolled CSF fluctuations.

### Role of endoscopic third ventriculostomy

Although CSF shunting remains the most common treatment for PTH, alternative surgical options are available. ETV has historically been considered as first therapeutic option in obstructive hydrocephalus (60), with the procedure being frequently preferred over shunts (61, 62) because of its long-term outcomes and a lower likelihood of re-operation and postoperative infections (63, 64).

The concept of ETV acting solely as an internal shunt does not fully account for its clinical effectiveness in forms of hydrocephalus without a clear anatomical obstruction, including PTH (65). Although the precise pathophysiological basis for ETV efficacy in communicating hydrocephalus remains incompletely understood, multiple clinical studies have demonstrated favorable outcomes in this setting (62, 66). Contemporary hydrodynamic models of hydrocephalus suggest that impaired intracranial compliance and abnormal transmission of pulsatile CSF forces, rather than simple flow obstruction, may contribute to hydrocephalus (67). In this setting, advanced imaging modalities, such as phase-contrast MRI for CSF flow quantification, diffusion tensor imaging for microstructural injury, and MR elastography for brain compliance, have been used to characterize the altered CSF dynamics and parenchymal properties in communicating hydrocephalus (68, 69). In particular, phase-contrast MRI enables non-invasive quantification of CSF flow dynamics, revealing that patients with communicating hydrocephalus exhibit increased aqueductal CSF flow velocities and volumes compared to healthy controls (70). Overall, these techniques revealed biomechanical instability at the brain-CSF interface, with loss of parenchymal stiffness and viscoelasticity facilitating ventricular enlargement even at normal or low intracranial pressures (71), shifting the paradigm from a purely CSF-centric to a brain-CSF interface model of disease. Within this framework, ETV may exert its therapeutic effect by modifying CSF dynamics, reducing intracranial pulse pressure, and alleviating compression of venous and capillary capacitance vessels, thereby interrupting the pathological cycle underlying communicating hydrocephalus (72, 73) and explaining ETV’s efficacy. Dynamic neuroimaging have also shown to that CSF flow velocities and volumes decrease after successful ETV, supporting the mechanism of restored CSF circulation following this procedure (62).

Historically, PTH has been considered a relative contraindication for ETV (65). Extensive meningeal and subarachnoid scarring occurring in PTH was thought to impair the systolic CSF pulsations required for ETV efficacy and to limit the expansion of subarachnoid spaces and cortical veins, thereby reducing intracranial compliance and cerebral perfusion (65, 74). Supporting this concept, Mohanty et al. demonstrated a correlation between the severity of basal cisternal arachnoid adhesions and poorer clinical outcomes (75). Despite these concerns, accumulating clinical evidence challenges the traditional reluctance to use ETV in PTH. A meta-analysis by De Bonis et al. reported clinical improvement in 93% of PTH patients treated with ETV, while Chrastina et al. found that 54.5% of patients with PTH or post-hemorrhagic hydrocephalus remained shunt-free with favorable Glasgow Outcome Scale scores. Together with emerging pathophysiological insights into CSF dynamics, these data support a role for neuroendoscopic treatment in selected PTH patients (60, 65).

In summary, although ETV outcomes remain dependent on hydrocephalus etiology and individual clinical characteristics, the procedure appears to confer meaningful benefit in a substantial proportion of adult patients (62), including PTH patients (60, 65). Nevertheless, the current evidence base is limited by small sample sizes and predominantly retrospective designs, underscoring the need for larger, prospective clinical trials to better define patient selection criteria and long-term outcomes.

### Cranioplasty and shunting: who comes first?

DC is a potentially lifesaving procedure commonly required in TBI patients to control refractory ICP increases. However, it is consistently associated with an increased risk of PTH and other secondary complications (1, 76, 77). Experimental studies and clinical evidence suggest that DC may promote ventriculomegaly and then hydrocephalus by disrupting physiological CSF dynamics (2, 3, 8, 78, 79).

Approximately 44% of severe TBI survivors develop ventriculomegaly, with true hydrocephalus occurring only in about one-third of cases (80). Quantitative assessment of CSF dynamics, such as the measurements of ICP, the resistance to CSF outflow (Rcsf), and CSF flow velocities, play a central role in the accurate diagnosis of hydrocephalus (1, 80, 81). In particular, ex-vacuo ventriculomegaly is characterized by normal CSF dynamics testing and represents passive ventricular enlargement secondary to brain parenchymal loss rather than active CSF accumulation; these patients typically show proportionate sulcal widening and normal parameters (80). In these cases, shunting is not required and the outcome is generally better than in hydrocephalic groups, though functional deficits from the primary brain injury persist (80). Post-traumatic normal-pressure hydrocephalus presents with ventriculomegaly, normal baseline ICP (<15 mmHg), but elevated Rcsf (>12–13 mmHg/mL/min), reflecting impaired CSF absorption despite normal resting pressure (80, 81); patients may be symptomatic, though presentation is often incomplete or delayed and imaging shows disproportionate ventriculomegaly relative to sulcal prominence, with tight high-convexity sulci and dilated Sylvian fissures (DESH pattern) (69, 82, 83). Post-traumatic high-pressure hydrocephalus is defined by ventriculomegaly with elevated baseline ICP (>15 mmHg), with or without elevated Rcsf (80), occurring acutely (within days) or subacutely (weeks to months) and presenting with signs of intracranial hypertension (9). The variety of the condition highlights the importance of appropriate diagnosis and patients’ selection to ensure a positive outcome of shunt surgery and avoid overtreatment.

Several DC-related technical factors have been identified as potential contributors to the development of PTH (50), such as the distance of the medial edge of the craniectomy from the midline (84), the size of the craniectomy (50, 85), the presence of subdural hygroma (86), and delayed cranioplasty (87). Many attempts have been made to provide a valuable explanation of the intrinsic correlation among these factors, DC and PTH. In particular, De Bonis et al. hypothesized that a craniectomy too close to the midline may alter CSF dynamics by determining an increase in the venous outflow during the diastolic phase, with a subsequent increase of extracellular fluid absorption and its accumulation in the subdural space. This mechanism may lead to interhemispheric subdural hygroma development and eventually ventricular enlargement (84). The size of the DC may, as well, affect the CSF pressure dynamics and induce inflammatory processes in the region of the Pacchioni granulations, promoting hydrocephalus (50). Nevertheless, a recent comprehensive review identified a wide range of modifiable and non-modifiable variables associated with DC-related hydrocephalus (88), suggesting that this condition may be more closely related to the severity and distribution of the primary brain injury rather than to any single technical parameter, and is therefore likely multifactorial.

Cranioplasty (CP) represents the standard reconstructive procedure following DC and serves not only to restore cranial protection but also to re-establish more physiological CSF circulation and cerebral blood flow dynamics (89). Despite these benefits, CP is associated with a substantial risk of complications, including subdural fluid collections, infection, hydrocephalus, seizures, sinking skin flap syndrome, and subdural hygromas, with reported complication rates of approximately 10–15% in large series and significantly higher rates in complex cases (4, 89, 90). Surgical timing and implant material are key determinants of CP outcome (89). A crucial finding from the recent meta-analysis by Chibbaro et al. is that surgical timing significantly influences complication rates. Recent data indicate that many CP-related complications are minor and manageable when identified early, and that standardized referral pathways, structured decision-making, and systematic follow-up can significantly reduce adverse event rates over time (89). Two main risk factors for complications were identified: the presence of a VPS at the time of CP and a sunken brain appearance on preoperative CT imaging, highlighting the particular vulnerability of shunt-dependent patients, in whom complication rates may exceed 50% (5, 89, 91).

Considerable uncertainty remains regarding the relationship between CP and PTH, with available evidence derived from heterogeneous observational studies (1). Some studies advocate for a protective role of early CP in PTH development (20) while others suggest the opposite result with early CP (15–30 days after DC) being associated with increased hydrocephalus risk and delayed procedures (>90 days) with lower hydrocephalus rates (6, 92). Additional investigations have failed to demonstrate a clear association between CP timing and PTH incidence (5), and hydrocephalus may persist despite CP and presumed restoration of CSF pulsatility (86). Technical and perioperative strategies—such as minimizing epidural dead space, optimizing CSF pressure and volume management, and individualizing shunt settings—may mitigate complications, although the supporting evidence remains largely observational (89).

The optimal sequencing of CP and shunting is similarly unresolved. A central issue is whether combined (one-stage) CP with VPS placement should be favored over a staged approach. Retrospective series and meta-analyses have yielded conflicting results: some studies report that simultaneous CP and VPS may reduce reoperations or hospital-acquired infections in selected cohorts (7–9, 93–95), whereas others describe higher overall complication rates, particularly subdural effusions, wound complications, and infections—especially when VPS placement precedes CP or when PTH is not clearly established (10, 11, 96–101). A recent meta-analysis including approximately 500–650 patients demonstrated that simultaneous CP and VPS placement nearly doubled the risk of overall complications compared with staged procedures, with a notably higher incidence of subdural effusions (approximately 14% vs. 5–6%), although differences in specific outcomes such as infection or hemorrhage were less consistent and sometimes lost statistical significance in sensitivity analyses (98). Classic series, including that of Heo et al., reported overall complication rates of 43% after DC, increasing to 56% in patients undergoing simultaneous CP and VPS, compared with 21% in those managed with staged procedures (91). Subsequent institutional series have largely confirmed these findings, reporting complication rates of 33–47% for one-stage procedures versus 9–29% for staged approaches (91, 97). Severe brain bulging at the time of CP has consistently emerged as a strong predictor of adverse outcomes and is widely considered a relative contraindication to simultaneous reconstruction and shunting (97, 98). A meta-analysis concluded that staged CP and VPS procedures might benefit from a potentially lower trend in complication rates. However, more studies are needed to clarify this, since many variables such as the differences in the CP transplantation materials, the degree of brain bulging, timing of surgery following the initial causative event, and the interval and sequence between CP and VPS have not been considered as factors influencing the data on reported complications (98).

These ongoing uncertainties prompted a recent European expert consensus initiative (102) addressing the diagnosis and surgical management of CP and PTH. The panel favored a staged strategy, performing CP first and reserving definitive shunt placement for patients with confirmed or highly probable PTH, primarily to avoid overtreatment of post-traumatic ventriculomegaly and to account for the limited diagnostic reliability of current tools prior to skull reconstruction (12, 102–104). Within this framework, dynamic clinical assessment and serial neuroimaging are emphasized over reliance on single radiological measurements. Commonly used indices such as Evans’ ratio >0.3 may be difficult to interpret in decompressed patients and were therefore considered of uncertain value. Instead, evolving neurological deficits, persistently elevated intracranial pressure, scalp fullness, and failure of neurological improvement over time are regarded as more reliable indicators of PTH, particularly when evaluated longitudinally (102).

In patients at high risk of shunt failure or in whom the diagnosis of PTH remains uncertain, especially medically fragile individuals, the panel recommends extending dynamic CSF studies and infusion testing after CP, as pre-reconstruction measurements of CSF outflow resistance are insufficient to guide definitive shunt decisions. A staged approach thus provides an opportunity to differentiate transient post-traumatic ventriculomegaly that may stabilize or improve after CP from true PTH requiring permanent CSF diversion (102). When shunting is ultimately indicated, programmable valves are considered the preferred option, as they allow adaptation to evolving CSF dynamics and help reduce the risk of chronic overdrainage, particularly in cases where ventriculomegaly does not necessarily reflect fixed hydrocephalus (105–108).

Taken together, current evidence indicates that DC is a major risk factor for the development of PTH, although its pathophysiology is multifactorial and reflects a complex interaction between primary injury severity, altered CSF dynamics, and surgical factors. The timing and sequencing of CP and CSF diversion should therefore be individualized, with an emerging consensus favoring staged CP followed by VPS placement only when clearly indicated, rather than simultaneous reconstruction and shunting. In the absence of definitive data and given persistent uncertainty regarding optimal diagnostic thresholds and surgical strategies, the adoption of structured institutional protocols, aligned with expert consensus recommendations and supported by systematic outcome monitoring, is strongly recommended to standardize care and improve long-term outcomes in this complex patient population (102).

### Conservative management

Attempts to treat hydrocephalus using small-molecule interventions have been documented since the early 1900s. These approaches were largely empirical, not supported by robust evidence, and did not achieve meaningful clinical success. Nowadays, the role of conservative treatments in the management of PTH is often limited to symptom control and used as temporary measures before definitive surgical treatment: one of the first pharmacologic attempts to treat hydrocephalus consisted of theobromine sodio salicylate, and glycerol, both used as diuretic agents. Currently, clinically used agents include osmotic and loop diuretics such as mannitol and furosemide, which both function via inhibition of CSF secretion, and are frequently used in combination. However, Mannitol is not advised for prolonged use beyond 72 h because of the risk of developing rebound intracranial hypertension. Other pharmaceutical options include Dexamethasone (which can be administered at 12–16 mg/day for 4–6 weeks), Acetazolamide (administered in a 100 mg/kg dose for up to 1 month), and Isosorbide, the latter serving as an effective adjunctive measure for temporary ICP control before shunt placement. Notably, the concurrent use of Acetazolamide and Furosemide has proved to be significantly superior to Acetazolamide alone in achieving normal ICP levels. Moreover, Atorvastatin therapy has been reported to be beneficial for hydrocephalus control, boosting the nervous system recovery by promoting angiogenesis. Overall, despite playing a fundamental role in PTH management, these drugs have not yet been able to treat the condition. Recent preliminary experimental research on animal models has explored the potential role of molecular targets in the pathogenesis of communicating hydrocephalus including blood clot resolution using fibrinolytics, modulation of thrombin and TGF-*β*-mediated fibrosis, chelation of iron and hemoglobin-induced oxidative injury, inhibition of red blood cell lysis and complement pathways, antioxidant strategies such as melatonin and VEGF inhibition, and glymphatic system impairment via circHIPK3 and c-Src kinase (109–111). Furthermore, emerging regenerative approaches, particularly stem cell transplantation using human umbilical cord blood–derived mesenchymal stromal cells, have shown promising neuroprotective and anti-inflammatory effects in preclinical and early clinical studies, especially in pediatric IVH-related hydrocephalus (111); also hypothermia seems to alleviate neurological dysfunction and attenuate hydrocephalus and pathological damage by improving the function and structure of the meningeal lymphatic system and may therefore have the potential to ameliorate IVH-induced hydrocephalus and neurological dysfunction (112). However, these results are still preliminary, and continuous research is necessary to develop compounds with more direct and selective properties that can offer long-term clinical benefits in the conservative management of PTH.

Collectively, while current conservative treatments remain largely supportive and temporary, ongoing treatment mainly relies on surgical options.

### Other innovative treatment options

Emerging techniques for the management of PTH aim to optimize CSF diversion while minimizing complication rates, reflecting a broader shift toward individualized and pathophysiology-driven treatment strategies. External ventricular drainage (EVD) remains a cornerstone in the acute management of critically ill patients with suspected or confirmed PTH, providing both temporary CSF diversion and ICP control as well as a dynamic diagnostic tool to assess CSF circulation and predict responsiveness to permanent shunting. However, prolonged use of conventional EVD is limited by the risks of infection, catheter obstruction, and the need for intensive monitoring.

In this context, long tunneled external ventricular drainage (LTEVD) has attracted growing interest as a means to reduce infection risk compared with standard short tunneled systems. A more advanced variant, laparoscopic temporary LTEVD (LT LTEVD), has recently been investigated specifically in the setting of PTH (11). In LT LTEVD, the distal end of the ventricular catheter, located at the level of the thorax or abdomen, is advanced approximately 15 cm into a self-made external catheter and secured by an inflatable balloon, creating a “barrier effect” intended to decrease retrograde infection. Once neurological status stabilizes and adequate CSF flow is confirmed, the balloon is deflated and the external catheter is removed, leaving the distal catheter in the abdominal cavity and thereby converting the temporary system into a standard VPS (113, 114). Preliminary results suggest that the outcomes of LTEVD in pediatric and adult neurosurgical populations appear comparable to conventional EVD, with a trend toward lower infection rates in some series, although these differences have not consistently reached statistical significance and existing data remain limited (11, 113). Arachnoid granulations and villi from 23 brains from subjects aged 9 to 84 years were examined post-mortem by serial sections with the light microscope and as whole or fractured preparations in the scanning electron microscope. The object of the study was to investigate the pathways within the arachnoid granulations by which cerebrospinal fluid drains from the subarachnoid space to the sinus endothelium. At the base of each granulation, a thin neck of arachnoid projects through an aperture in the dural lining of the sinus and expands to form a core of collagenous trabeculae and interwoven channels. An apical cap of arachnoid cells, about 150 μm thick, surmounts the collagenous core, and channels extend through the cap to reach subendothelial regions of the granulation. Channels within the granulation are lined by compacted collagen and may contain macrophages. Following recent subarachnoid hemorrhage, erythrocytes are found in the channels, suggesting that the channels are in continuity with the subarachnoid space and are CSF drainage pathways. The cap region of the granulation is only attached to the endothelium over an area 300 μm in diameter; the rest of the granulation core is separated from the endothelium by a subdural space and a fibrous dural cupola. Scanning electron microscopy reveals an intact endothelial surface to the granulations with small perforating venous channels present on the apex of some granulations. The differences between human arachnoid granulations and arachnoid villi in animals are discussed, together with preliminary observations regarding the transition of villi into granulations in man (2). Given the paucity of robust evidence, LTEVD and LT LTEVD should currently be used in a carefully selected, individualized manner, guided by anticipated drainage duration, patient risk profile, and institutional expertise, but they represent promising adjuncts in the PTH armamentarium.

In parallel, neuromodulation strategies have emerged as a novel avenue for adjunctive treatment in severe TBI, with potential implications for patients with PTH and disorders of consciousness (115). Experimental work in animal models of explosive brain injury has shown that vagus nerve stimulation (10 V, 5 Hz, 20 min) can reduce brain edema, decrease pro inflammatory cytokines (TNF *α*, IL 1β), and increase the anti-inflammatory cytokine IL 10, suggesting a neuroprotective effect mediated through modulation of neuroinflammation. Building on these findings, Hakon et al. demonstrated the feasibility and safety of transcutaneous vagus nerve stimulation (tVNS) in patients with severe TBI, applying stimulation for 4 hours daily over 8 weeks in individuals with disorders of consciousness; three of five patients showed improved consciousness with minimal side effects, indicating that tVNS may enhance brain plasticity and support recovery of consciousness. Nonetheless, the current evidence base is very limited, and further controlled studies are required to define optimal stimulation protocols, identify responders, and clarify whether such approaches can meaningfully influence long term outcomes in PTH and severe TBI (116). Although these emerging techniques—advanced tunneled drainage systems and neurostimulation—are not yet widely implemented and remain supported by low to moderate quality evidence, they illustrate an important ongoing evolution toward more tailored, pathophysiology-oriented management of PTH. As data accumulate, integration of these strategies with established surgical and rehabilitative pathways may help refine risk–benefit profiles, reduce complications, and improve outcomes in this highly vulnerable patient population.

### PTH treatment, long-term prognosis and rehabilitation

Long-term neurological recovery, functional outcome, and rehabilitation needs after TBI are drastically influenced by PTH. Prognosis following shunt surgery depends on accurate diagnosis, appropriate patient selection, timely CSF diversion, and access to intensive multidisciplinary rehabilitation programs.

PTH frequently emerges during the post-acute rehabilitation phase and can be difficult to detect, often manifesting as stagnation or deceleration of neurological recovery, with conventional neuroimaging alone often being insufficient to distinguish ex vacuo ventricular dilation from true hydrocephalus, which can be normal pressure or high-pressure. Despite these diagnostic challenges, the effectiveness of shunt placement in PTH is well supported in the literature, with earlier shunting being consistently associated with greater functional gains during rehabilitation (19) and delayed shunting associated with more complex and prolonged recovery (14). Although PTH is associated with unfavorable functional outcomes and prolonged rehabilitation trajectories (1, 14), patients treated with shunt surgery can still achieve substantial functional improvement and long-term outcomes comparable to those of non-shunted post-DC patients, albeit with a greater need for prolonged and intensive rehabilitation (1, 8). Patients with PTH are often medically fragile, present multiple comorbidities, and require intensive rehabilitation programs to translate improved CSF circulation into meaningful functional recovery and better quality of life.

In a large cohort of patients with severe acquired brain injury who developed hydrocephalus, most individuals demonstrated improvements in both cognition and disability during inpatient rehabilitation. Mean Disability Rating Scale (DRS) scores improved from vegetative-level severe disability at admission to severe disability with high dependence at discharge, and nearly 90% of patients showed measurable cognitive gains (14). Longer lengths of stay were more frequently observed in younger patients, likely reflecting greater investment in rehabilitation and the impact of intercurrent complications, as well as in patients with poorer functional status at discharge (14). Prognostic models, such as that proposed by Wang et al. for patients with severe disorders of consciousness, further emphasize that younger age, higher GCS scores, less severe hydrocephalus, and earlier shunting independently predict more favorable outcomes, underscoring the importance of early identification and targeted intervention (117). Outcome assessment in PTH, particularly in patients with disorders of consciousness, remains challenging, and multiple clinical scales, including the Glasgow Outcome Scale (GOS), DRS, Functional Independence Measure (FIM), and neurobehavioral measures, have been employed to capture the benefits of shunt surgery and rehabilitation at follow-up. Literature lacks a direct comparison of the functional outcomes among the different surgical options. However, ETV and VPS seem to have comparable success rates (defined as GOS 4 or 5 without shunt) with a rate of good outcomes (GOS 4–5) of ETV being slightly lower than with VPS (65). In a recent case series, a decrease in disability score (from 21.2 upon admission to 14.5 at discharge, which means the transition from vegetative state to severe disability with high dependence and low social reintegration) was seen in patients with severe acquired brain injury treated with VPS (14).

In parallel to clinical functional assessment, advanced imaging biomarkers are increasingly integrated into clinical decision-making: functional MRI and MR ventriculography are used to assess cerebral blood flow and subarachnoid space patency and to predict shunt responsiveness or evaluate ETV results, while CT perfusion offers a more widely available modality to quantify cerebral blood flow, blood volume, and mean transit time, with studies showing perfusion abnormalities in hydrocephalus and identifying age and CT-based severity as predictors of post-shunt outcome. CT and shuntography also play a crucial role in detecting shunt malposition or malfunction, directly informing revision strategies and influencing long-term prognosis; incorporation of such imaging markers into standardized clinical protocols may refine patient selection and optimize timing of intervention (9).

Finally, the burden of PTH and surgical management extends severely to family caregivers (9) who commonly report difficulties maintaining a normal life (10). Unmet needs commonly reported include better access to reliable expertise concerning shunt management and malfunction recognition, adequate psychological support, and financial assistance (10).

In summary, PTH represents a major and potentially modifiable determinant of long-term outcome after severe TBI. While timely shunt surgery can restore CSF dynamics and support neurological recovery, PTH remains associated with increased clinical vulnerability, prolonged rehabilitation, and complex functional trajectories. Optimal outcomes therefore depend not only on early and accurate diagnosis and appropriate surgical intervention, but also on sustained, intensive multidisciplinary rehabilitation and longitudinal monitoring (1, 9, 10, 14, 111, 117).

## Conclusion

PTH is a serious but treatable complication of severe TBI, with outcomes highly dependent on early diagnosis and timely intervention. Surgical CSF diversion remains the cornerstone of treatment requiring an individualized approach to shunt selection and valve management to accommodate post-traumatic CSF dynamics and account for the systemic fragility and multi-organ involvement. While VPS remains the gold standard, VAS and LPS options should be prioritized as primary alternatives when anatomical constraints, such as extensive abdominal scarring or compromised cranial access, complicate standard placement. ETV, though effective, remains a secondary option when shunting is contraindicated. The integration of programmable valves with gravitational units is essential for managing the complex hydrodynamic shifts occurring during postural rehabilitation, significantly reducing over-drainage risks compared to fixed-pressure systems. DC increases the risk of PTH and current evidence favors a staged approach over simultaneous procedures to maintain pressure stability and minimize complications. Although research into conservative strategies is progressing, these interventions currently serve only as temporary supportive measures. Early surgical intervention is essential, offering the best opportunity to mitigate long-term disability and optimize functional recovery in this complex patient population.
